# Endoscopic findings in patients with chronic bloating/abdominal distension and the effect of the transition from the Rome III to Rome IV criteria: a multicenter cross-sectional study

**DOI:** 10.1186/s12876-026-04634-7

**Published:** 2026-01-24

**Authors:** Jiandi Wu, Zhiyue Xu, Tao Bai, Xiaohua Hou, Jun Song

**Affiliations:** Department of Gastroenterology, Union Hospital, Tongji Medical College, Huazhong University of Science and Technology, 1277 Jiefang Road, Wuhan, 430022 China

**Keywords:** Bloating/distension, Upper and lower gastrointestinal symptoms, Positive endoscopic findings, Rome diagnostic criteria, Functional gastrointestinal disorder

## Abstract

**Background:**

Bloating/abdominal distension is a common gastrointestinal symptom. However, the abnormal endoscopy results associated with bloating/abdominal distension and its influencing factors in outpatients remain unclear.

**Methods:**

This multicenter cross-sectional study was conducted in 100 tertiary care hospitals nationwide and involved the use of a mobile applet to complete questionnaires to record the medical history of outpatients with bloating/distension. This was followed by an analysis of the upper and lower gastrointestinal endoscopic detection results and the factors influencing them. Finally, the transition from the Rome III to Rome IV criteria was explored to assess changes in the diagnostic landscape in patients with chronic bloating/distension.

**Results:**

A total of 1481 patients with chronic bloating/distension were included. The rate of abnormal endoscopic examination of upper gastrointestinal tract (GI) symptoms was greater than that of lower GI symptoms (16.6% vs. 11.5%, *P* < 0.05). Logistic regression revealed that alarm signs were the only risk factor associated with abnormal upper GI findings (*P* = 0.040; OR = 1.753; 95% CI: 1.026–2.995), and a disease duration greater than 6 months was a protective factor for positive lower GI endoscopy findings (*P* = 0.036; OR = 0.590; 95% CI: 0.360–0.967). Comparative analysis revealed diverging diagnostic trends under the Rome IV criteria. Specifically, PDS diagnoses decreased by 6.1% and IBS by 7.7%, whereas FC, U-FDB and FAB/D increased by 1.3%, 2.7% and 10.5%, respectively. EPS rates remained stable, with DGBI overlap diagnoses increasing by 1.2%.

**Conclusions:**

The positive endoscopic finding of upper GI bloating/distension was greater than that of lower GI, and the risk factors associated with positive findings were alarm symptoms but had limited sensitivity/specificity. The transition from the Rome III to Rome IV criteria changed the diagnostic landscape for patients with chronic bloating.

**Supplementary Information:**

The online version contains supplementary material available at 10.1186/s12876-026-04634-7.

## Background

Bloating and abdominal distension are common gastrointestinal symptoms. Bloating refers to a subjective sensation (e.g., feelings of abdominal fullness, pressure, or discomfort), whereas abdominal distension represents objective, visible or measurable enlargement of the abdomen. In English-speaking countries, the prevalence of bloating/distension as a symptom ranges from 11% to 30% [1–4]. It is a heterogeneous condition caused by different etiologies, including nongastrointestinal diseases (such as diabetes mellitus and hypothyroidism) [5] as well as organic and functional disorders of the gastrointestinal tract. Disorders of gut–brain interaction (DGBI), formerly known as functional GI disorders (FGIDs), commonly cause bloating/distension in clinical practice, including functional dyspepsia, irritable bowel syndrome, functional constipation, and functional bloating/abdominal distension [6–8]. Organic diseases such as gastrointestinal tumors and chronic liver disease are also common [9].

Bloating/distension extensively affects the quality of life of patients. Bloating/distension appears to be associated with higher levels of depression and anxiety as well as lower levels of mental health and physical health [10]. Constant outpatient follow-up, the administration of various medications, and various tests impose a heavy financial burden on individuals as well as on society [7]. Over the past few years, various clinical studies have pointed to bloating/distension as important and bothersome complaints in functional gastrointestinal disorders [11]. However, bloating/ distension may be a manifestation of organic disease, and the underlying etiology should be considered first in the differential diagnosis [12]. Currently, chronic bloating/distension as a unique presenting symptom—and, in particular, its distinctiveness from classic dyspepsia or irritable bowel syndrome (IBS) in guiding endoscopic decision-making—remain incompletely understood. Although high-quality studies have documented endoscopic yields in cohorts with presumed FGID/DGBI, the specific prevalence of endoscopic abnormalities among patients whose primary complaint is chronic bloating has not been clearly established. Notably, previous endoscopic analyses in FGID/DGBI populations have focused on heterogeneous symptom clusters such as dyspepsia or IBS and have failed to investigate whether chronic bloating in isolation is associated with a distinct endoscopic profile—a critical unmet clinical question that requires further clarification. Therefore, we conducted a multicenter observational study with the aim of investigating the clinical characteristics of outpatients with chronic bloating/distension as well as the detection rate and associated factors of abnormal endoscopy findings according to different sites of bloating/distension.

Duration, symptoms, and frequency as the primary basis for diagnosing DGBI according to the Rome criteria. The transition from the Rome III to Rome IV criteria halved the prevalence of IBS but increased the prevalence of functional constipation and functional diarrhea, mainly by increasing the frequency threshold for the diagnostic criteria for IBS and removing “abdominal discomfort” [13]. However, the effects of the transition from the Rome III to Rome IV criteria on diagnostic redistribution, particularly the diagnostic influence of patients with chronic bloating, are unclear. Therefore, our study seeks to further compare the applications of the Rome III and IV criteria in the bloating/distension population.

The aims of this study were as follows: (i) to assess abnormal endoscopic detection of upper and lower gastrointestinal symptoms in the bloating/distension population as well as associated factors, thus providing guidance for gastroenterology clinics; (ii) to compare the applications of the Rome III and IV criteria in the bloating/distension population.

## Methods

### Study design and participants

This multicenter cross-sectional study was conducted from November 1, 2021, to October 31, 2022, in 100 tertiary care hospitals nationwide and used a mobile applet to complete questionnaires (including initial visit and follow-up visit supplementary data) to record the disease history and examination of outpatients presenting with complaints of bloating/distension. All initial visit patients were requested appropriate endoscopy, unless the duration of bloating/distension was greater than 2 years and endoscopic findings had been obtained in the past 2 years. The inclusion criteria were as follows: (1) age ≥ 18 years; (2) chronic bloating/distension (onset time ≥ 1 month) as the main complaint; (3) endoscopic findings corresponding to the site of bloating/distension obtained within the past 2 years; (4) willingness to participate in the research project. The exclusion criteria were as follows: (1) history of abdominal surgery; and (2) diseases causing bloating/distension (cholecystitis, gallstones, fatty liver with abnormal liver function, hepatitis, cirrhosis, chronic pancreatitis, pelvic inflammatory disease, diabetes mellitus, etc.).

### Procedures and content

A total of 1–2 physicians (one associate chief physician and above and one attending physician) were selected from each hospital separately, and the participating physicians were trained on how to complete the questionnaire before the study began. Data were collected and managed uniformly through a small program supervised by the project leader, with a database set up, logical proofreading and passwords, and regular review of the data.

The questionnaire mainly included baseline information, past medical history, surgical history, endoscopic findings, liver and renal function blood markers, accompanying symptoms, alarming features and content related to bloating/distension (frequency, degree, location and type of bloating/distension). The degree of bloating/distension was recorded by applying a bloating/distension scale of 1–10. Gastrointestinal bleeding, persistent vomiting and weight loss, as described in the Rome IV criteria, were considered alarming features [14]. According to the location of the symptoms and the characteristics of the accompanying symptoms, they were categorized as upper gastrointestinal symptoms, lower gastrointestinal symptoms, and overlapping upper and lower gastrointestinal symptoms. This classification is primarily based on the Rome criteria to distinguish between upper gastrointestinal symptoms such as functional dyspepsia, a condition that includes early satiety as a core feature of postprandial distress syndrome, and lower gastrointestinal symptoms such as irritable bowel syndrome, which is characterized by bowel-related symptoms such as altered stool frequency or consistency [14]. Upper gastrointestinal symptoms included abdominal pain or bloating in the upper and middle portions of the abdomen, early satiety or postprandial fullness and were not accompanied by a change in bowel habits or constipation. Lower gastrointestinal tract symptoms included abdominal pain or bloating/distension in the lower and middle parts of the abdomen, constipation or a change in bowel habits, not accompanied by early satiety or postprandial fullness. Upper and lower GI overlap was noted based on the following features: (i) the site of bloating/distension was the whole abdomen or upper and lower abdomen; (ii) upper symptoms and lower symptoms overlap (the presence of early satiety or postprandial fullness and constipation or bowel-related bloating/pain or abdominal distension). The above categorization was used to explore the rate of positive endoscopic detection of symptom types.

### Disease diagnosis using Rome III and Rome IV criteria

According to the current study, bloating/distension is often associated with functional dyspepsia [15] and functional bowel disorders [11]. Disease categorization in the chronic bloating population was based on the diagnostic criteria of Rome III and Rome IV, with reference to endoscopic findings [16, 17].

### Statistical analysis

We mainly apply descriptive statistical analysis methods to represent categorical data and continuous data as percentages and means, respectively. Count data are described as the number of cases and percentages (%). Prior to final patient enrollment, we excluded 9 patients whose datasets were incomplete (key data loss rate: 0.6%, 9/1490). The extremely low missing data rate (< 1%) minimized the impact on sample representativeness and result reliability. Univariate and multifactorial logistic regression analyses were used to assess the independent predictors of abnormal endoscopic results. During multivariate regression analysis, we assessed model fit using the Hosmer–Lemeshow goodness-of-fit test (all P values > 0.05, indicating satisfactory model fit) and evaluated multicollinearity among independent variables using variance inflation factor (VIF) analysis (all VIF values < 5, confirming the absence of significant multicollinearity). The SPSS 25.0 software package was used for statistical analysis, and a two-sided P value < 0.05 was considered to indicate statistical significance.

## Results

**Fig. 1:**
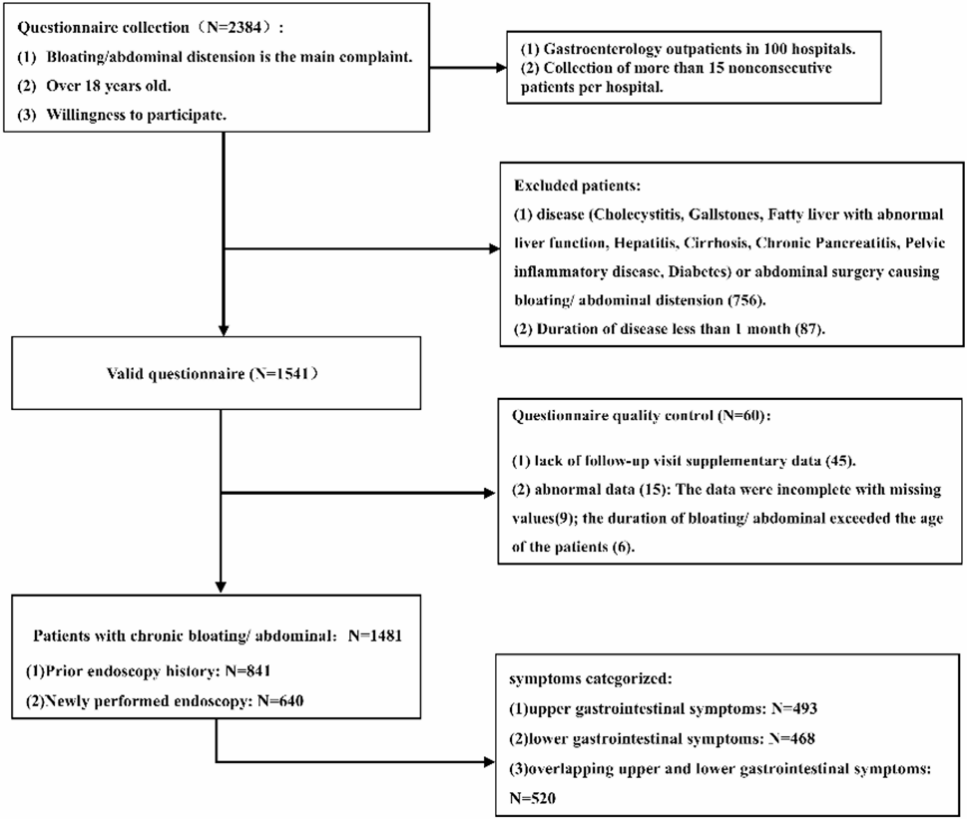
Flowchart of the research

The number of patient questionnaires collected was 2384, of which 1541 met the inclusion criteria. In total, 60 patients were subsequently excluded based on quality control: (1) 45 patients lacked questionnaires for follow-up visits; and (2) 15 were completed with abnormal data (9 incompletely filled, 6 with bloating duration > patient age). Finally, 1481 patients were included in our research (Fig. 1, flow chart).

### Distribution of baseline data and clinical characteristics

Among the patients with chronic bloating/distension, the proportion of females was greater than that of males (52.1%, males: 47.9%), with an average age of 47.4 ± 13.2 years (Table 1), and the majority of patients were nonsmokers (88.3%) and nonalcohol drinkers (93.7%).

**Table 1:** General characteristics of patients with chronic bloating/distension

Characteristic	*N* = 1481
Genders	
Males, *n* (%)	710 (47.9%)
Females, *n* (%)	771 (52.1%)
Age (years)	47.4 ± 13.2
BMI (kg/㎡)	23.0 ± 3.4
Area	
Central	350 (23.6%)
Eastern	360 (24.3%)
Northern	357 (24.1%)
Southern	414 (28.0%)
Marriage	
Unmarried	134 (9.0%)
Married	1323 (89.3%)
Divorced/widowed	24 (1.6%)
Level of education	
Primary school	233 (15.7%)
Middle or high school	659 (44.5%)
College or above	589 (39.8%)
Smoking status	
Never/occasionally	1308 (88.3%)
Quit smoking	33 (2.2%)
Frequent	140 (9.5%)
Drinking	
Never/occasionally	1388 (93.7%)
Quit drinking	12 (0.8%)
Frequent	81 (5.5%)

The probabilities of alarm signs and other symptoms among patients with bloating/distension were 7.8% and 83.3%, respectively (Table S1). Alarm signs in descending order of percentages were weight loss (5.5%), hematochezia (1.6%), persistent vomiting (0.6%) and hematemesis (0.5%). Other symptoms included postprandial fullness (56.2%), early satiety (40.2%), abdominal pain (34.0%), defecation (31.9%), constipation (15.8%) and poor appetite (8.0%). The results of the study revealed that 64.7% of the patients experienced moderate (3–6 points) to severe (7–10 points) bloating/distension, whereas the onset time of bloating/distension in patients was predominantly 1–3 months (62.4%). Patients with bloating/distension had a high frequency of episodes, with the proportion of patients with episodes occurring ≥ 1 day/week reaching 79.4%.

### Endoscopic findings in patients with different types of symptoms

**Fig. 2:**
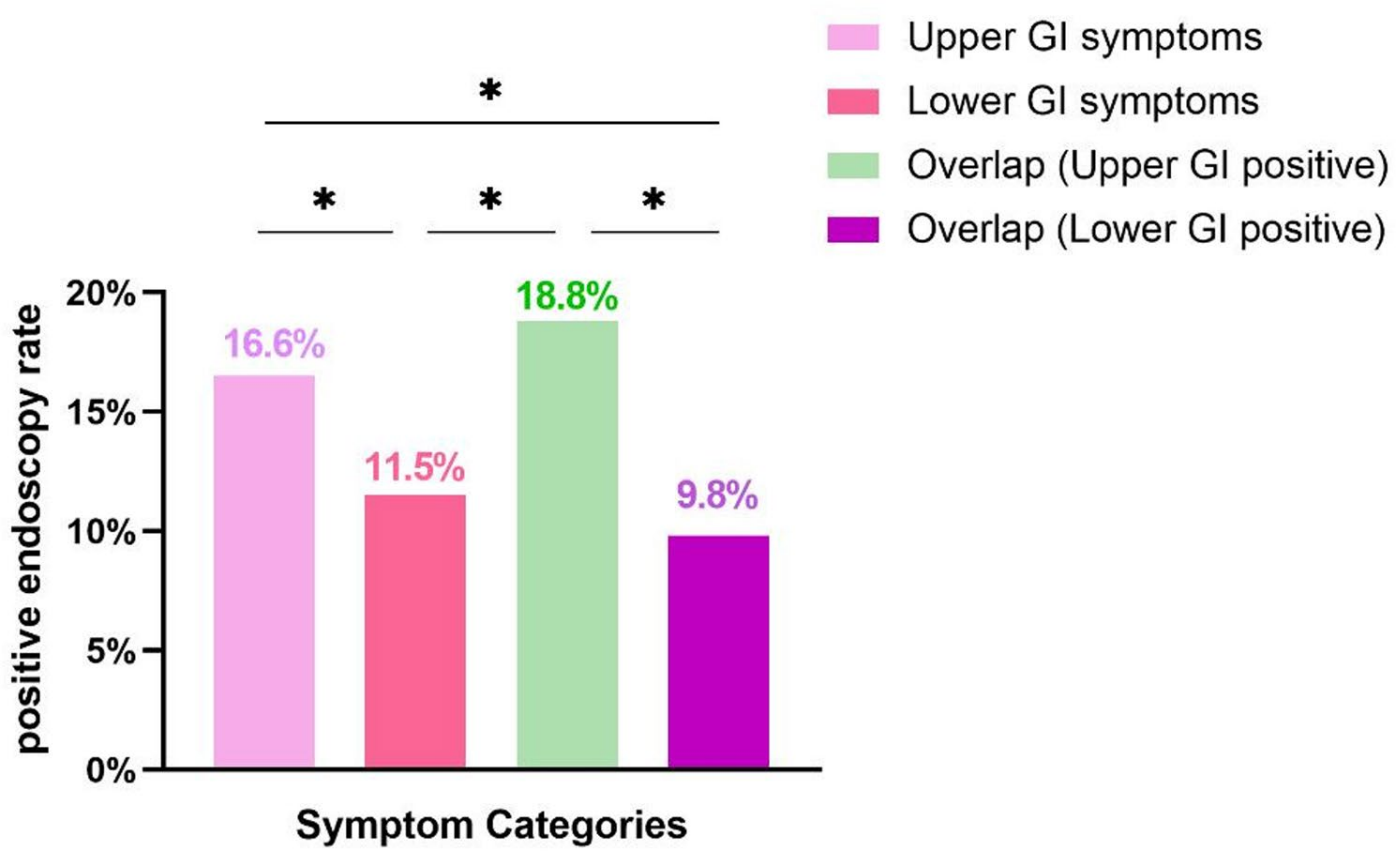
Abnormal endoscopic findings in patients with chronic bloating/distension

Details of the abnormal endoscopic results are shown in Table 2. The 1481 patients with bloating/distension were categorized as having only upper gastrointestinal symptoms, only lower gastrointestinal symptoms, and overlapping upper and lower gastrointestinal tracts, comprising 493, 468, and 520 patients, respectively (Table 3). The rate of abnormal endoscopic examination of upper GI symptoms was greater than that of abnormal detection of lower GI symptoms (16.6% vs. 11.5%, *P* < 0.05), and the rate of abnormal endoscopic detection of upper GI symptoms was also greater than that of lower GI symptoms in overlapping patients (18.8% vs. 9.8%, *P* < 0.05). A comparison of the results is shown in Fig. 2. To exclude selection bias arising from patients with bloating who underwent prior endoscopy, we also performed a comparative analysis between the total cohort and the cohort excluding patients with prior endoscopy. No substantial differences in detection rates were noted between the two cohorts (Table S2).

**Table 2:** Endoscopic abnormal findings in patients with chronic bloating/distension

Type of symptom	Endoscopic findings	
	Nontumor	Tumor
Only upper GI symptoms	42CAG 12RE 20PU 1BE 2EG 1HG 1AG 1CAG + PU 1CAG + RE	2GT
Only lower GI symptoms	26IME 13FMIC 7MAI 4MC 3UC	1 CT
Overlapping upper and lower GI symptoms	36CAG 14RE 21PU 1BE 3EG 1AG 2CAG + RE 1RE + PU 18IME 9FMIC 4MAI 1MC 1UC 6CAG + IME 2 CAG + FMIC 2PU + IME 2RE + IME 1RE + FMIC 2PU + IME	3GT 2CT

*CAG* Chronic atrophic gastritis, *RE* Reflux esophagitis, *PU* Peptic ulcer, *BE* Barrel’s esophagus, *GT* Gastric tumor, *EG* Esophagitis, *HG* Hemorrhagic gastritis, *AG* Acute gastritis, *IME* Isolated mucosal erythema, *FMIC* Focal mild inflammatory changes (edema/spotty erosion), *MAL* Minor aphthous lesions (non-IBD), *UC* Ulcerative colitis, *CT* Colorectal tumors, *MC* Melanosis coli

**Table 3:** Endoscopic findings in patients with chronic bloating/distension

Type of symptom	Endoscopic findings		Positive rate
	Negative	Positive	
Only upper GI symptoms	411	82	16.6% (82/493)
Only lower GI symptoms	414	54	11.5% (54/468)
Overlapping upper and lower GI symptoms	380	Only upper GI 82	Upper GI 18.8% (98/520) Lower GI 9.8% (51/520)
		Both upper and lower 16	
		Only lower GI 35	

*GI* Gastrointestinal tract

### Factors associated with positive endoscopic detection in the gastrointestinal tract

Univariate and multivariate logistic regression analysis of positive upper gastrointestinal endoscopic findings revealed that alarm signs were the only independent risk factor (*P* = 0.040, OR = 1.753, 95% CI: 1.026–2.995), and poor appetite was the only independent protective factor (*P* = 0.015, OR = 0.432, 95% CI: 0.220–0.849) (Table 4 and S3). One-way regression analysis of positive endoscopic findings revealed that a disease duration greater than 6 months was a protective factor for positive detection of lower GI endoscopy results compared with a duration of 1–3 months (*P* = 0.036; OR = 0.590; 95% CI: 0.360–0.967), and other factors were not relevant (Supplementary Table S4).

**Table 4:** Factors related to positive endoscopic findings of the upper GI tract (multifactorial logistic regression analyses)

Variable	β	S. E.	Wald test	*P*	OR	95% CI
Sex (female)	0.270	0.173	2.452	0.117	1.310	0.934–1.838
Age	-0.001	0.007	0.027	0.870	0.999	0.986–1.012
BMI	0.012	0.024	0.262	0.609	1.012	0.966–1.062
Bloating/distention Score (0–10)	0.016	0.047	0.119	0.730	1.016	0.926–1.110
Poor appetite	-0.839	0.344	5.937	0.015	0.432	0.220–0.849
Overlapping lower GI symptoms	0.150	0.167	0.804	0.370	1.162	0.837–1.613
Alarm symptoms	0.561	0.273	4.241	0.040	1.753	1.026–2.995

*GI* Gastrointestinal tract, *β* Beta (regression coefficient), *S. E* Standard error, *Wald* Wald χ² (Wald chi-square), *P* P value, *OR* Odds ratio, *95% CI* 95% Confidence interval

### Results of Rome III and Rome IV criteria for the diagnosis of patients with abdominal distension

**Fig. 3:**
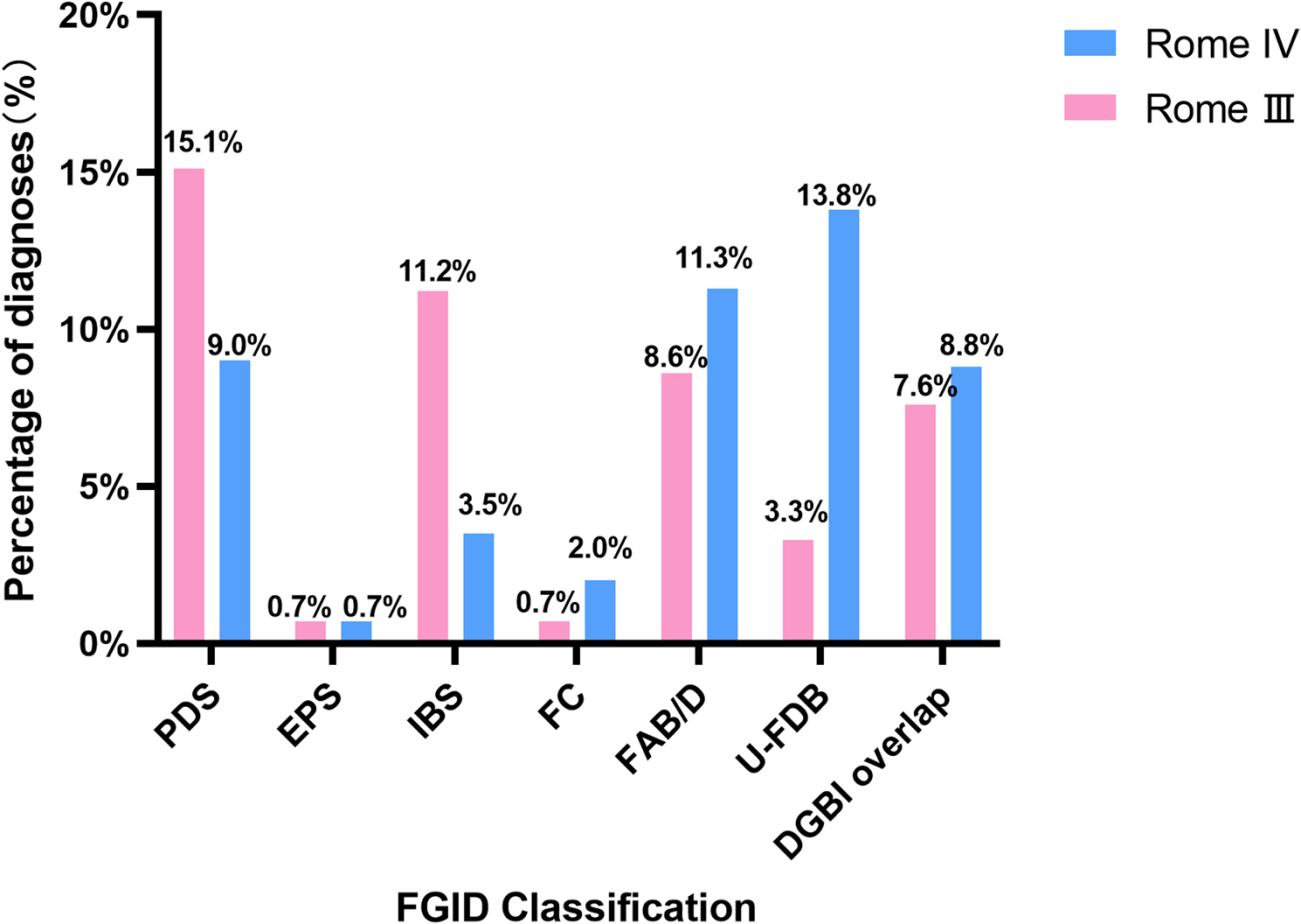
Results of Rome III and Rome IV criteria for the diagnosis of patients with abdominal distension. PDS, postprandial distress syndrome; EPS, epigastric pain syndrome; IBS, irritable bowel syndrome; FC, functional constipation; U-FDB, unspecified functional bowel disorder; FAB/D, functional abdominal bloating/distension; DGBI, disorders of gut–brain interaction

The diagnostic impact of Rome III and Rome IV adjustments in patients with bloating/distension were unclear. Therefore, we performed Rome III and Rome IV diagnostics for patients with negative endoscopic findings. The comparative analysis revealed notable shifts in diagnostic patterns between the Rome IV and Rome III criteria (Fig. 3; Table 5). Functional dyspepsia (FD) subtypes showed diverging trends, with postprandial distress syndrome (PDS) diagnoses decreasing by 6.1% and epigastric pain syndrome (EPS) remaining stable. In contrast, IBS diagnoses demonstrated a substantial 7.7% reduction. Conversely, several disorders markedly increased, including functional constipation (FC) by 1.3%, unspecified functional bowel disorder (U-FDB) by 10.5%, and functional abdominal bloating/ distension (FAB/D) by 2.7%. Notably, the prevalence of disorders of gut–brain interactions overlapping with gastrointestinal symptom overlap (DGBI overlap) diagnoses increased by 1.2%, reflecting enhanced recognition of comorbid presentations using the updated criteria.

**Table 5:** Results of Rome III and Rome IV criteria for the diagnosis of patients with bloating/distension

Rome	PDS	EPS	IBS	FC	FAB/D	U-FDB	DGBI overlap
Rome Ⅲ	224 (15.1%)	11 (0.7%)	166 (11.2%)	10 (0.7%)	127 (8.6%)	49 (3.3%)	112 (7.6%)
Rome IV	134 (9.0%)	11 (0.7%)	52 (3.5%)	30 (2.0%)	168 (11.3%)	205 (13.8%)	130 (8.8%)
Relative quantity	-90 (-6.1%)	0 (0%)	-114 (-7.7%)	20 (1.3%)	41 (2.7%)	156 (10.5%)	18 (1.2%)

*DGBI overlap* Disorders of gut–brain interaction overlap, *PDS* Postprandial distress syndrome, *EPS* Epigastric pain syndrome, *IBS* Irritable bowel syndrome, *FC* Functional constipation, *U-FDB* Unspecified functional bowel disorder, *FAB/D* Functional abdominal bloating/ distension, *DGBI* Disorders of gut–brain interaction

## Discussion

Bloating/distension are common and bothersome clinical symptoms in outpatients [1, 18]. This study was the first to explore the following endoscopic findings in a bloated population: prevalent chronic atrophic gastritis (CAG), reflux esophagitis (RE), and peptic ulcer (PU) in upper GI symptoms, and other and unspecified noninfectious gastroenteritis and colitis (OUNGC) in lower GI symptoms, including isolated mucosal erythema (IME), focal mild inflammatory changes (FMIC) and minor aphthous lesions (MAL). In patients with bloating/distension, the rate of endoscopic positivity for upper gastrointestinal symptoms was higher than that for lower gastrointestinal symptoms, and the independent risk factor was alarm symptoms but had limited sensitivity/specificity. Conversely, the only protective factor for a positive endoscopy for lower gastrointestinal symptoms was a disease duration greater than 6 months and no associated risk factors. After the Rome III to Rome IV criteria were adjusted, the diagnostic landscape changed for patients with chronic bloating/distension.

A systematic review revealed that gastroscopic findings in asymptomatic and symptomatic patients were 18.7% and 32.0% in Europe and 74.3% and 76.8% in China, respectively. The higher prevalence in China may be related to the unexpectedly high incidence of gastroduodenal erosive disease (49.9%) [19]. Our previous study revealed that patients with functional gastrointestinal symptoms were more likely to have positive gastroscopic findings, with a positive rate of 31.8% [20]. This study revealed a positive gastroscopy rate of 16.5–18.8% in patients with upper gastrointestinal symptoms of bloating/distension. A possible reason for our relatively low percentage of positive results was that upper abdominal-related bloating/distension is more often associated with functional diseases, and its positive endoscopic results were similar to the results of the asymptomatic population. Several studies have reported that the positive predictive value of both upper gastrointestinal tract-specific alarm symptoms and nonspecific alarm symptoms in predicting upper gastrointestinal tract cancers is low [19, 21], suggesting that alarm symptoms may be associated with organic upper gastrointestinal tract disorders rather than cancer. None of the five patients with stomach tumors in this study had associated alarm symptoms, and two of them had a disease duration of > 3 months and a bloating/abdominal severity score of 3–7. Therefore, some tumors may present as nonspecific. A study from a rural population of Sindh, Pakistan, revealed that positive gastroscopy results were more likely to be associated with upper gastrointestinal alarm symptoms such as dysphagia, heartburn, hematemesis, and vomiting [22], and the results of our study were similar. A study from China on GI symptoms and gastroscopy findings also revealed that alarm symptoms were risk factors for positive gastroscopy findings (*P* < 0.001, OR = 2.87; 95% CI: 2.02–4.08) [23]. Our study suggests that alarm symptoms are associated with positive gastroscopy results in patients with epigastric bloating but have limited sensitivity/specificity, which is consistent with results in populations with other functional gastrointestinal symptoms, but the positive detection rate is comparable to that in asymptomatic patients. Biologically, alarm symptoms such as unexplained weight loss, gastrointestinal bleeding and persistent vomiting are well-established markers of organic upper gastrointestinal pathology, including peptic ulcer disease and gastric neoplasia [24], reflecting structural damage or dysfunction of the upper GI tract mucosa. Our findings align with those of prior epidemiological studies showing that alarm symptoms independently predict endoscopic positivity for organic disease and are linked to pathological processes rather than functional disorders. Conversely, poor appetite emerged as a protective factor for upper GI positivity in our study, likely because postprandial distress syndrome, a subtype of functional dyspepsia, is characterized by early satiety and poor appetite. These symptoms are driven by gut–brain axis dysregulation, such as impaired gastric accommodation and visceral hypersensitivity, rather than structural mucosal damage, although rare cases of concurrent organic disease do exist. Further research is warranted to validate these findings. In addition, we determined that overlapping lower GI symptoms did not increase the proportion of positive endoscopic detections of upper GI symptoms. Moreover, we reported that the most common abnormal endoscopic findings in upper gastrointestinal bloating patients were chronic atrophic gastritis, reflux esophagitis, and peptic ulcers. A longitudinal study of the disease spectrum and natural history of patients with abdominal distension conducted in China also revealed that peptic ulcers were the primary factor in the organic disease of bloating/distension [25], but chronic atrophic gastritis and reflux esophagitis were not mentioned, which may be related to the use of multiple screening methods and the use of appropriate etiologic classification, and our study focuses on endoscopic findings.

Bloating/distension are usually considered secondary concomitant symptoms among outpatients [26, 27] and are distributed among different etiologies, and studies focusing on patients with lower gastrointestinal tract abdominal distension are rare. As a result, there are no clear conclusions regarding the detection of colonoscopy in patients with bloating/distension. In patients with lower gastrointestinal symptoms, our results revealed that the positivity rate of colonoscopy ranged from 9.8% to 11.5%, which is similar to the results of another study that reported a colonoscopy positive rate of 9.9% for DGBI symptoms [20]. In addition, a colonoscopy study that included 646 patients with functional bowel disorders also revealed a 12% prevalence of organic disease, including 6.2% with inflammatory bowel disease (IBD), 2.9% with microscopic colitis, and 3.1% with colorectal cancer [28]. We found that among patients with bloating/distension, the most common colonoscopy finding was OUNGC, accounting for 9.3% (92/988) of the cases. These abnormalities were subclassified as follows: (1) isolated mucosal erythema (*n* = 56, 5.7%); (2) minor aphthous lesions (non-IBD, *n* = 25, 2.5%); and (3) focal mild inflammatory changes (*n* = 11, 1.1%). In contrast, only three cases of colorectal tumors were detected in this patient subgroup. The three patients all had a duration of 1–3 months, including bloating in one case and abdominal distension and postprandial fullness in the other two, with a bloating score ranging from 3 to 6 and a frequency of 1 day/week, 2–3 days per week and daily, respectively. Therefore, none of the three patients met the criteria for a diagnosis of a DGBI. Moreover, one study revealed that 98% of patients who received a diagnosis of DGBI had warning symptoms with no significant specificity for positive detection [28]. Moreover, our study did not find an association between alarm symptoms and abnormal colonoscopy findings in patients with lower gastrointestinal bloating/distension, suggesting that the role of alarm symptoms in lower gastrointestinal bloating/distension differs from that of upper gastrointestinal symptoms. We also reported that lower gastrointestinal bloating/distension that overlapped upper gastrointestinal symptoms did not increase abnormal colonoscopy findings in the lower gastrointestinal tract, suggesting that bloating/distension is more of a physiologic change in function, such as visceral hypersensitivity, among other causes. Many articles have focused on the association of bloating/distension with functional gastrointestinal disorders [29, 30], and relevant guidelines have noted that bloating/distension as a symptom can be present in a wide range of patients with functional gastrointestinal disorders [31]. Bloating in FGIDs is driven by three core mechanisms: abnormal intestinal gas retention and maldistribution [32], impaired gastric accommodation to meals [33], and visceral hypersensitivity to intestinal distension [34]. These pathways interact synergistically to define “functional” phenotypes, distinguishing them from the organic causes of bloating [35]. In clinical practice, patients with bloating/distension must have a sufficient duration of illness to be diagnosed with DGBI. However, is it possible that a shorter duration of illness may result in the detection of more abnormal results? Few studies have explored such conditions. The results of this study revealed that a duration of > 6 months is a protective factor for abnormal colonoscopy results compared with a duration of 1–3 months, suggesting that the duration of disease influences abnormal colonoscopy findings. This finding may be attributed to the fundamental differences in the pathophysiological mechanisms between these two categories of diseases. Specifically, the development of functional bowel disorders is primarily associated with intestinal motility disorders, visceral hypersensitivity, or gut microbiota dysbiosis—pathological changes that are typically chronic and nonprogressive [36]. In contrast, organic pathologies are characterized by irreversible mucosal damage or tumor formation, and their symptoms tend to present as acute onset or rapid progression.

Our study revealed that more than 60% of patients were afflicted by moderate to severe bloating/abdominal, as well as episodes with a frequency of ≥ 1 day/week in 79.4% of cases, which is similar to the results of a previous bloating-based study conducted in the United States [37]. Currently, the transition from the Rome III to Rome IV diagnostic criteria has been studied in relation to both the IBS population and the screening population [13, 38], but there are fewer studies on the impact on the classification of patients with bloating/distension. Similar to population-based screening findings, the adoption of the Rome IV criteria in patients with bloating-related symptoms resulted in a marked decrease in IBS diagnoses (-7.7%) and an increase in functional constipation diagnoses (+ 1.3%). Concurrently, the number of PDS diagnoses decreased significantly (-6.1%), whereas the number of FAB/D cases increased only modestly (+ 2.7%). A significant 10.5% increase in U-FDB diagnoses was observed, attributable to the stricter frequency thresholds of the Rome IV criteria for FAB/D. The significant reductions in diagnoses of IBS and PDS can be attributed to the stricter diagnostic criteria introduced in Rome IV. For IBS, the definition was narrowed to require recurrent abdominal pain (rather than the broader “abdominal discomfort/pain” in Rome III) associated with defecation or changes in stool frequency/form, alongside an increased minimum symptom frequency (≥ 1 day/week for 3 months vs. ≥3 days/month in Rome III), which excluded patients with nonpain-related bowel symptoms or subthreshold pain frequency. For PDS, Rome IV mandated the presence of both postprandial fullness and early satiety (vs. “one or more symptoms” in Rome III) and specified symptom persistence for ≥ 3 days/week (a criterion absent in Rome III), thereby reclassifying patients with isolated postprandial fullness or infrequent symptoms out of the PDS category. Concomitantly, the marked increase in U-FDB diagnoses directly stems from the reclassification of subthreshold cases previously categorized under IBS or PDS. As a “catch-all” category for FGIDs that fulfill general FGID criteria but do not meet subtype-specific requirements (Rome IV, 2016), U-FDB currently encompasses patients with persistent functional bowel symptoms (e.g., bloating without concurrent abdominal pain or isolated early satiety) who no longer qualify for IBS or PDS because of the stricter thresholds of Rome IV criteria. This shift is particularly pronounced for patients with subthreshold bloating/distension. Rome IV introduced quantitative frequency thresholds (e.g., ≥ 1 day/week for IBS, ≥ 3 days/week for PDS) that were either absent or less stringent in Rome III, excluding individuals with clinically present symptoms that did not meet subtype-specific frequency criteria (e.g., bloating for 2 days/week, insufficient for PDS; abdominal distension without weekly pain, insufficient for IBS) and reassigning these patients to U-FDB. Our findings align with established epidemiological trends reported in Rome IV-focused studies. Specifically, Tornkvist et al. (2025) reported a 6% reduction in IBS prevalence globally following the adoption of Rome IV, driven by the revised pain and frequency criteria [39]. Simadibrata et al. (2025) reported a diagnosis rate increase in U-FDB diagnoses in Southeast Asia, attributable to the reclassification of subthreshold cases from specific FGID subtypes [40]. These consistencies validate the clinical relevance of our observed diagnostic redistribution, highlighting the broader impact of Rome IV criteria on the epidemiology and classification of FGID. This reclassified cohort may necessitate enhanced therapeutic vigilance and precision-tailored pharmacotherapy to address their revised diagnostic status.

Our study has several limitations. First, our study was a cross-sectional study conducted in an outpatient setting, where the diagnosis of patients’ diseases was based mainly on patients’ symptoms and examination data and was diagnosed by the receiving physician, which may be heterogeneous. In addition, the designed questions were not very comprehensive and lacked questions about patients’ psychological status, activity, and treatment costs; however, these questions have been described in other studies [1, 37]. Second, the localization of bloating symptoms is often subjective and imprecise, which may introduce potential misclassification bias. To mitigate this limitation, we adopted standardized symptom assessment tools aligned with the Rome IV diagnostic criteria to define upper GI symptoms (e.g., early satiety, epigastric pain or bloating) and lower GI symptoms (e.g., bowel movement-related pain, altered stool habits) rather than relying solely on patients’ self-reported “symptom location”. Third, several potential biases and data gaps warrant consideration. For example, recall bias may be inherent in the collection of symptom frequency and severity scores because of their reliance on participants’ self-reported recall. In addition, the absence of *Helicobacter pylori* (*H. pylori*) data precludes an evaluation of its potential impact on the prevalence of CAG, RE, and PU, and the lack of standardized endoscopic reporting protocols across the 100 participating centers may have introduced heterogeneity in diagnostic data and reduced the comparability of results. Moreover, the number of patients we included was still relatively small in the context of a large epidemiologic survey study, and the results were limited. Finally, because the Rome IV diagnostic criteria for functional diarrhea require that the patient have “no abdominal discomfort”, functional diarrhea was not explored in this study (bloating population).

## Conclusion

In summary, our study revealed endoscopic findings in upper and lower GI bloating/distension patients. Positive endoscopic detection of upper GI bloating was higher than that of lower GI, and the associated risk factor was alarm symptoms. However, this factor had limited sensitivity/specificity. Disease duration > 6 months was an independent protective factor for positive endoscopic detection in the lower gastrointestinal tract. Moreover, the overlap of symptoms in the upper and lower GI tracts did not influence the likelihood of positive detection in either tract. After the transition from the Rome III to Rome IV criteria, the diagnostic landscape for patients with chronic bloating/distension changed.

## Supplementary Information

Supplementary Material 1.

Supplementary Material 2.

## Data Availability

Data were obtained from questionnaires completed at the initial and follow-up patient visits, and the data supporting these results will not be made available to the public because of privacy and ethical constraints. Interested scholars can contact the corresponding author for access.

## Abbreviations

GI: Gastrointestinal tract
PDS: Postprandial distress syndrome
EPS: Epigastric pain syndrome
IBS: Irritable bowel syndrome
FC: Functional constipation
U-FDB: Unspecified functional bowel disorder
FAB/D: Functional abdominal bloating/ distension
DGBI: Disorders of gut–brain interaction
FGID: Functional gastrointestinal tract disorders
IBD: Inflammatory bowel disease
CAG: Chronic atrophic gastritis
RE: Reflux esophagitis
PU: Peptic ulcer
BE: Barret’s esophagus
GT: Gastric tumor
EG: Esophagitis
HG: Hemorrhagic gastritis
AG: Acute gastritis
OUNGC: Other and unspecified noninfectious gastroenteritis and colitis
UC: Ulcerative colitis
CT: Colorectal tumors
MC: Melanosis coli
IME: Isolated mucosal erythema
FMIC: Focal mild inflammatory changes
MAL: Minor aphthous lesions
