# Supplementary material for: Endoscopic findings in patients with chronic bloating/abdominal distension and the effect of the transition from the Rome III to Rome IV criteria: a multicenter cross-sectional study
Source: BMC Gastroenterol. 2026 Jan 24;26:134. doi: 10.1186/s12876-026-04634-7 (PMC12911186; doi:10.1186/s12876-026-04634-7)
Supplement: Supplementary file 1 — Supplementary Material 1. [file 12876_2026_4634_MOESM1_ESM.docx]

**supplementary material：**

Table S1. Distribution of symptoms in patients with chronic bloating/abdominal distension.

| **classifications** | N=1481 |
| --- | --- |
| **Alarm signs**  Hematemesis  Hematochezia  Weight loss  Persistent vomiting  **Other symptoms**  Poor appetite  Postprandial fullness  Early satiety  Abdominal pain  Constipation  Defecation-related | 115 (7.8%)  7 (0.5%)  24 (1.6%)  82 (5.5%)  9 (0.6%)  1366 (83.3%)  118 (8.0%)  833 (56.2%)  595 (40.2%)  504 (34.0%)  234 (15.8%)  473 (31.9%) |
| **Bloating/ distention Score (0-10)**  1-3  4-6  7-10 | 523(35.3%)  777 (52.5%)  181 (12.2%) |
| **Course of bloating/ Abdominal distention** (months)  1-3  4-6  >6 | 924 (62.4%)  253 (17.1%)  304 (20.5%) |
| **Frequency of bloating /Abdominal distention**  1 day/ month  2-3 days/ month  1 day/ week  2-3 days/ week  4-6 days/ week  7 days/ week | 91 (6.1%)  215 (14.5%)  141 (9.5%)  439 (29.6%)  185 (12.5%)  410 (27.7%) |

Table S2. Comparison of endoscopic abnormality findings rates between the total cohort and the cohort excluding patients with prior endoscopy.

| **Type of symptom** | **Total sample**  **(n=1841)** | **Cohort excluding patients with prior endoscopy (n=640)** | **P value** |
| --- | --- | --- | --- |
| **Only Upper GI symptoms** | 16.6% (82/493)  95% CI (13.51% ~ 20.19%) | 17.9% (32/179)  95% CI (12.99% ~ 23.90%) | p = 0.656 |
| **Only Lower GI symptoms** | 11.5% (54/468)  95% CI (8.95% ~ 14.75%) | 7.11% (15/211)  95%CI (4.36% ~ 11.40%) | p = 0.1030 |
| **Overlapping upper and lower GI symptoms** | **Upper GI**: 18.8% (98/520)  95% CI (15.72% ~ 22.42%)  **Lower GI**: 9.8% (51/520)  95% CI (7.52% ~ 12.68%) | **Upper GI**: 14.0% (35/250)  95% CI (10.25% ~ 18.83%)  **Lower GI**: 5.6% (14/250)  95% CI (3.36% ~ 9.43%) | **Upper GI**:  p = 0.096  **Lower GI**  p = 0.063 |

Table S3. Other related factors of the positive endoscopic findings of upper GI tract (univariate analysis).

| **Variable** | ***β*** | ***S. E.*** | ***Walds*** | ***P*** | **OR** | **95% *CI*** |
| --- | --- | --- | --- | --- | --- | --- |
| **Marriage**  Unmarried  Married  Divorced/widowed | -  0.049  0.078 | -  0.296  0.832 | -  0.027  0.009 | -  0.870  0.925 | -  1.050  1.081 | 1  0.587 – 1.877  0.212 – 5.519 |
| **Level of education**  Primary school  Middle or high school  College or above | -  -0.150  -0.180 | -  0.232  0.234 | -  0.420  0.589 | -  0.517  0.443 | -  0.836  0.246 | 1  0.546 – 1.356  0.528 – 1.322 |
| **Smoking status**  Never/occasionally  Quit smoking  Frequent | -  -0.746  -0.313 | -  0.748  0.313 | -  0.993  0.625 | -  0.319  0.429 | -  0.474  0.781 | 1  0.109 – 2.056  0.423 – 1.441 |
| **Drinking**  Never/occasionally  Quit drinking  Frequent | -  -19.668  0.076 | -  16408.711  0.361 | -  0.000  0.044 | -  0.999  0.834 | -  0.000  1.079 | 1  0.000  0.532 – 2.190 |
| **Course of bloating/ Abdominal distention**  1-3/ months  4-6/ months  >6/ months | -  0.034  -0.228 | -  0.215  0.190 | -  0.025  1.437 | -  0.876  0.231 | -  1.034  0.796 | 1  0.679– 1.575  0.548 – 1.156 |
| **Frequency of bloating /Abdominal distention**  1 day/ month  2-3 days/ month  1 day/ week  2-3 days/ week  4-6 days/ week  7 days/ week | -  0.079  0.336  0.243  0.620  0.020 | -  0.457  0.465  0.411  0.434  0.414 | -  0.029  0.522  0.350  2.042  0.002 | -  0.864  0.470  0.554  0.153  0.962 | -  1.082  1.399  1.275  1.859  1.020 | 1  0.441 – 2.652  0.563 – 3.476  0.570 – 2.854  0.794 – 4.349  0.453 – 2.298 |
| **Postprandial fullness** | -0.195 | 0.208 | 0.876 | 0.349 | 0.823 | 0.548 – 1.237 |
| **Early satiety** | -0.201 | 0.166 | 1.467 | 0.226 | 0.818 | 0.591 – 1.132 |
| **Abdominal pain** | 0.197 | 0.165 | 1.419 | 0.234 | 1.218 | 0.881 – 1.683 |

Table S3. Related factors of the positive endoscopic findings of lower GI tract (univariate analysis).

| **Variable** | ***β*** | ***S. E.*** | ***Walds*** | ***P*** | **OR** | **95% *CI*** |
| --- | --- | --- | --- | --- | --- | --- |
| **Sex (female)** | 0.233 | 0.208 | 1.254 | 0.263 | 1.263 | 0.839 – 1.900 |
| **Age** | 0.004 | 0.008 | 0.253 | 0.615 | 1.004 | 0.989 – 1.019 |
| **BMI** | -0.001 | 0.030 | 0.001 | 0.976 | 0.999 | 0.942 – 1.059 |
| **Marriage**  Unmarried  Married  Divorced/widowed | -  0.132  0.765 | -  0.367  0.654 | -  0.130  1.365 | -  0.718  0.243 | -  1.142  2.148 | 1  0.556 – 2.344  0.596 – 7.745 |
| **Level of education**  Primary school  Middle or high school  College or above | -  0.272  0.441 | -  0.338  0.340 | -  0.645  1.684 | -  0.422  0.194 | -  1.312  1.554 | 1  0.676 – 2.545  0.799 – 3.023 |
| **Smoking status**  Never/occasionally  Quit smoking  Frequent | -  -0.968  -0.078 | -  1.028  0.339 | -  0.886  0.053 | -  0.346  0.818 | -  0.380  1.081 | 1  0.051 – 2.850  0.557 – 2.100 |
| **Drinking**  Never/occasionally  Quit drinking  Frequent | -  -19.052  0.456 | -  12710.133  0.378 | -  0.000  1.455 | -  0.999  0.228 | -  0.000  1.578 | 1  0.000  0.752 – 3.313 |
| **Frequency of bloating /Abdominal distention**  1 day/ month  2-3 days/ month  1 day/ week  2-3 days/ week  4-6 days/ week  7 days/ week | -  0.157  0.401  0.433  0.838  0.230 | -  0.543  0.557  0.501  0.531  0.514 | -  0.084  0.520  0.750  2.486  0.200 | -  0.773  0.471  0.387  0.115  0.655 | -  1.170  1.494  1.543  2.312  1.258 | 1  0.404 – 3.390  0.502 – 4.448  0.578 – 4.114  0.816 – 6.551  0.459 – 3447 |
| **Course of bloating/ Abdominal distention**  1-3/ months  4-6/ months  >6/ months | -  -0.019  -0.528 | -  0.260  0.252 | -  0.005  4.380 | -  0.942  0.036 | -  0.981  0.590 | 1  0.589 – 1.634  0.360 – 0.967 |
| **Bloating/ distention Score (0-10)** | -0.060 | 0.059 | 1.052 | 0.305 | 0.942 | 0.839 – 1.056 |
| **Poor appetite** | -0.542 | 0.475 | 1.304 | 0.253 | 0.581 | 0.229 – 1.475 |
| **Overlapping upper GI symptoms** | -0.179 | 0.207 | 0.755 | 0.385 | 0.836 | 0.557 – 1.253 |
| **Alarm symptoms** | 0.262 | 0.341 | 0.591 | 0.442 | 1.300 | 0.666 – 2.538 |
| **Abdominal pain** | 0.097 | 0.219 | 0.194 | 0.660 | 1.101 | 0.717 – 1.693 |
| **Constipation** | -0.239 | 0.256 | 0.868 | 0.352 | 0.788 | 0.477 – 1.302 |
| **Defecation-related** | -0.095 | 0.207 | 0.210 | 0.647 | 0.909 | 0.606 – 1.365 |
